# Duplication cyst of pyloroduodenal canal: a rare cause of neonatal gastric outlet obstruction: a case report

**DOI:** 10.1186/1757-1626-2-42

**Published:** 2009-01-12

**Authors:** Vijai D Upadhyaya, Punit K Srivastava, Richa Jaiman, Ajay N Gangopadhyay, Dinesh K Gupta, Shiv P Sharma

**Affiliations:** 1 Department of pediatric surgery IMS, BHU, Varanasi, India

## Abstract

**Background:**

A 21 day old male child presented with non bilious vomiting and abdominal mass.

**Case presentation:**

This case is reported because pyloroduodenal duplication cysts are an extremely rare congenital anomaly, whose clinical presentation often mimics those of hypertrophic pyloric stenosis. Ultrasound examination showed cystic mass at pyloric region and barium study was suggestive of extrinsic mass compressing the pyloric region. A laparotomy, a tense cystic mass was present at the pyloroduodenal junction (PDC) which was resected and end to end anastomosis was done. Patients followed an uneventful recovery and doing well.

**Conclusion:**

The clinical and radiological analysis can reveal configurational changes consistent with a large extrinsic mass rather than muscular hypertrophy and can lead to accurate preoperative diagnosis.

## Background

Gastrointestinal duplications are observed in 1 of every 4500 autopsies, predominantly in white males. The small intestine is the most frequent site involved, whereas gastric, duodenal, rectal, and thoracoabdominal involvement is relatively rare. Pyloroduodenal duplication cyst is an extremely rare and only two cases have been reported in English literature one by Tihanaski [1] and another by Y. Hamada [2]. Pyloroduodenal duplication cyst presents as gastric outlet obstruction and is very difficult to differentiate it clinically with infantile hypertrophic pyloric stenosis [IHPS] because it present with palpable abdominal mass and non bilious vomiting which may or may not be projectile. We are reporting a male neonate who presented with nonbilious projectile vomiting and palpable mass in right upper abdomen which mimics like IHPS but on exploration was found to be a case of pyloroduodenal duplication cyst.

## Case presentation

**Figure 1:**
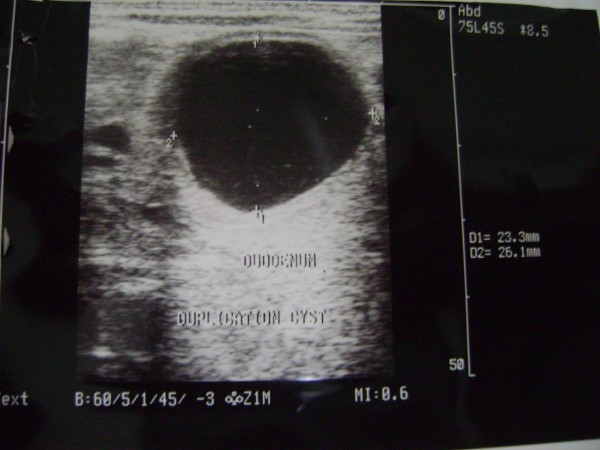
**USG showing the cystic lesion at pyloroduodenal region**.

**Figure 2:**
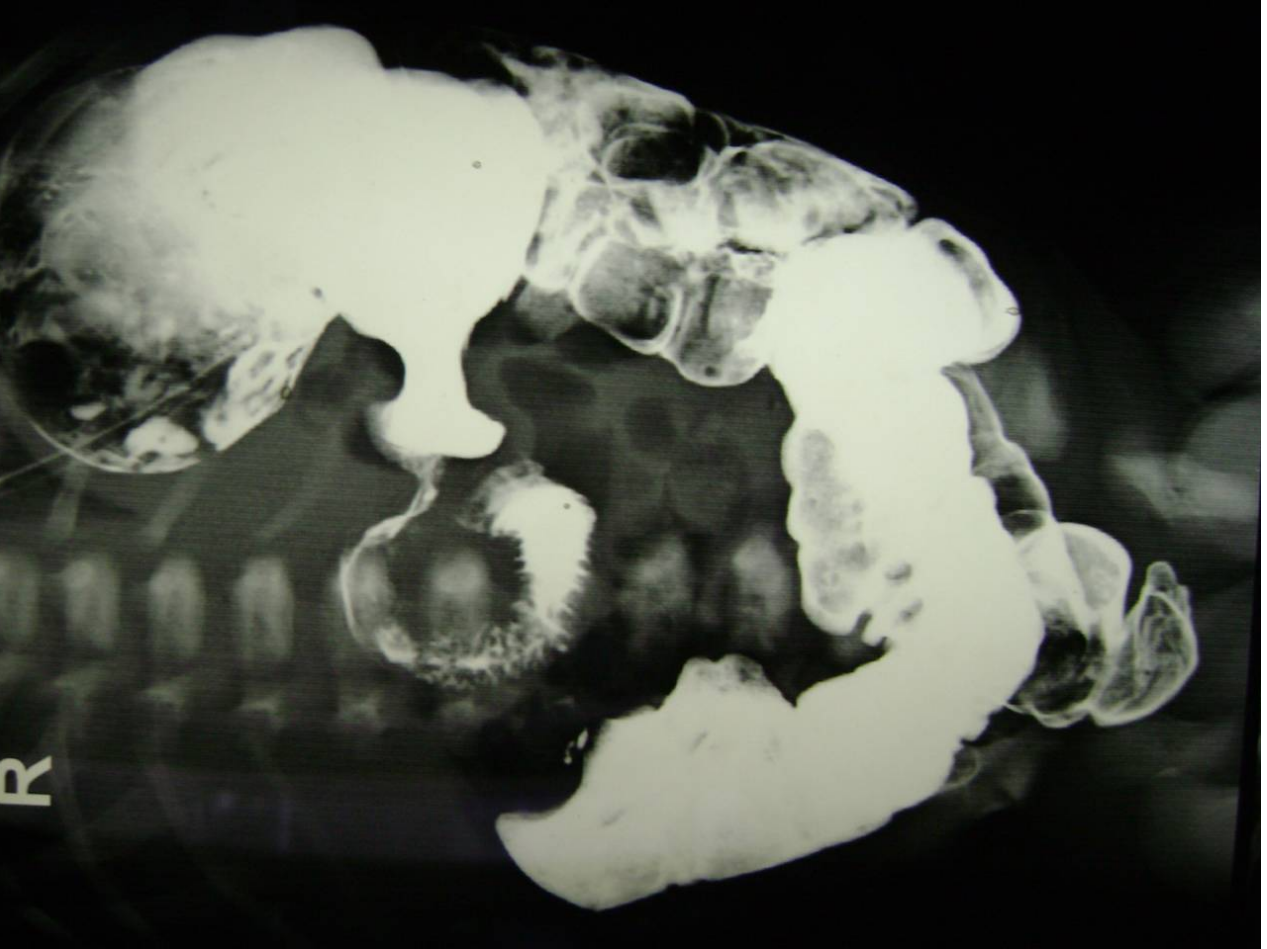
**Upper GI contrast study showing lesion at pyloroduodenal region**.

**Figure 3:**
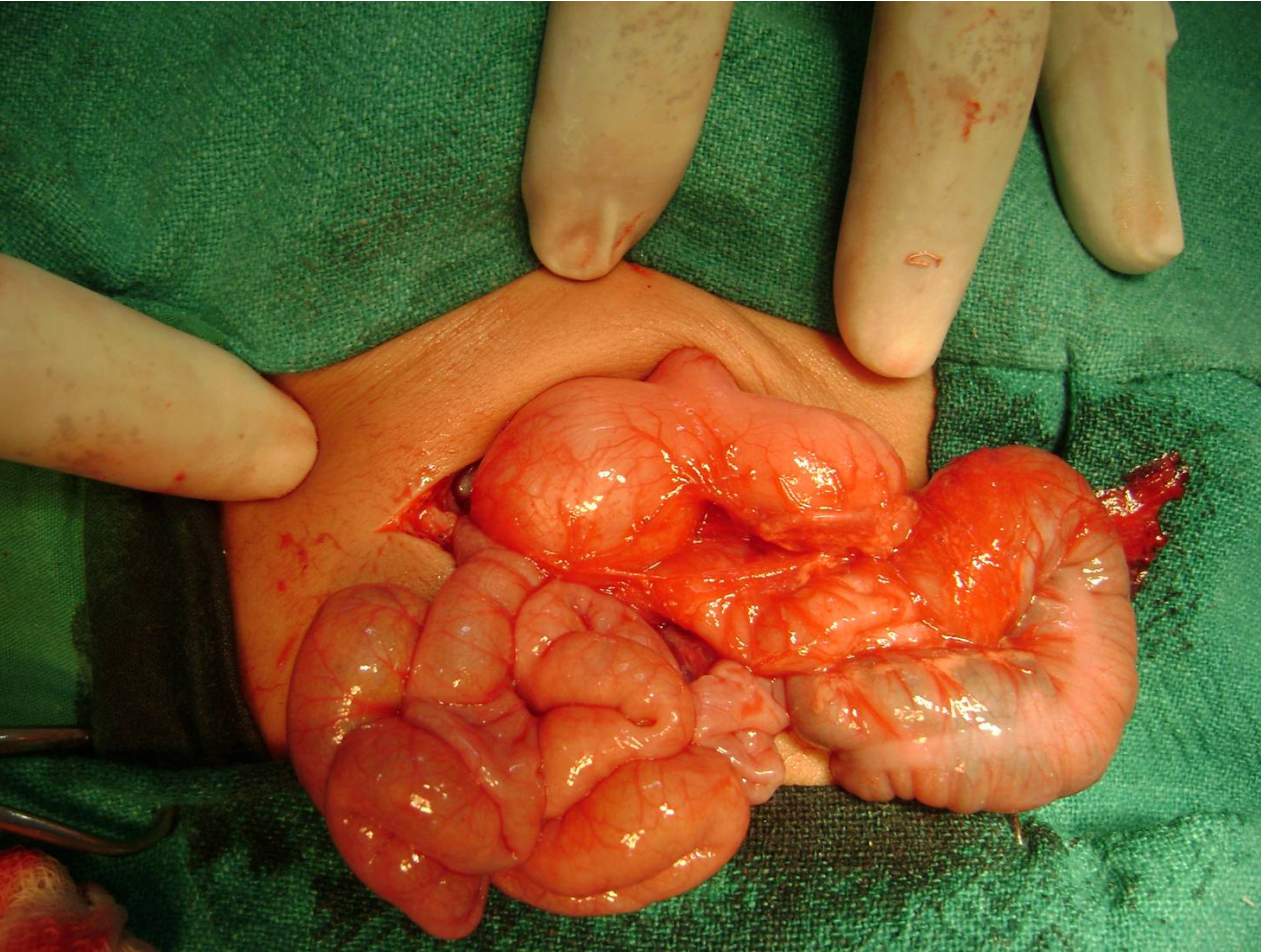
**Perioperative photograph showing distended stomach with cystic lesion at pyloroduodenal region**.

A 21 days old male neonate weight 3.2 kg, was presented to us with history of recurrent non bilious projectile vomiting after taking feeds. On physical examination a mobile mass of 3 cm × 2.5 cm was palpable in right upper abdomen. Ultrasonography of abdomen revealed a cystic mass in the pyloro-duodenal region measuring 2.6 × 2.3 cm (Figure 1). Upper gastrointestinal barium study was suggestive of mass at the pyloroduodenal junction (Figure 2). Clinical diagnosis was infantile hypertrophic pyloric stenosis [IHPS] but radiological investigations were suggestive of cystic duplication of pyloroduodenal junction. Patients was prepared for elective operation and on exploration, there was a cystic mass of about 4 × 3 cm at the pyloro-duodenal junction (Figure 3). The cystic mass was excised (because it was away from the second part of duodenum and easily excisable without complication) and end to end anastomosis between pylorus and duodenum was done. The excised cyst contains yellowish, serous fluid which has high CEA (carcinoembryonic antigen) level of 482 ng/m but it is non-specific. On histological examination, the common wall; had muscular layer and mucosal lining of duodenum, without evidence of gastric or pancreatic mucosa.

## Discussion

Pyloroduodenal duplication cysts (PDC) are an extremely rare congenital anomaly, presents with symptoms of gastric outlet obstruction and palpable mass which is clinically indistinguishable from IHPS. Enteric duplications can occur anywhere along the GI tract, but are most commonly found in the ileum and are perhaps rarest along the pyloric channel [1] and represent 2.2% of all gastric duplications [3]. The earliest case of a juxtapyloric cyst was reported by Ramsay [4] in 1957. Till now only eight cases have been reported so far in English literature [5]. Pyloroduodenal duplications most commonly present with projectile vomiting early in the neonatal period because of the partial gastric outlet obstruction secondary to compression of the pyloric channel. The accurate diagnosis of this lesion can be made by CT scan, but it cane be easily suspected on the basis of ultrasound and upper GI contrast study. Pre-operative assessment is mandatory when patient present with non-bilious projectile vomiting with easily palpable lump in right upper abdomen. Surgical options should be individualized to each case. These vary from simple excision to pyloro-antrectomy. In previous cases enucleation of the cyst was done where as we have removed the lesion in Toto because of the risk of development of malignancy in untreated duplication cyst [6]. We are reporting this case because a when a male neonate presents with nonbilious projectile mass with easily palpable lump in right upper abdomen the diagnosis of pyloric duplication cyst should be ruled out before operation to avoid surgical complication.

## Consent

Written informed consent was obtained from the parents of the patient for publication of this case report and accompanying images. A copy of the written consent is available for review by the Editor-in-Chief of this journal.

## Competing interests

The authors declare that they have no competing interests.

## Authors' contributions

SPS and PS operated the patient and reviewed the literature. VDU and RJ was the main writer of the manuscript. ANG and DKG moderated the manuscript.

All authors read and approved the final manuscript

## References

[B1] Tihansky DR Sukarochana K Hanrahan JB Pyloroduodenal duplication cyst Am J Gastroenterol 1986 81 189 191 3953534

[B2] Hamada Y Inoue K Hioki K Pyloroduodenal duplication cyst: case report Pediatr Surg Int 1997 12 2/3 194 5 10.1007/BF01349999 9156858

[B3] Murty TV Bhargava RK Rakas FS Gastroduodenal duplications J Pediatr Surg 1992 27 515 7 10.1016/0022-3468(92)90351-7 1522469

[B4] Ramsay GS Enterogenous cyst of stenosis the stomach simulating Hypertrophic pyloric stenosis Br J Surg 1957 44 632 633 10.1002/bjs.18004418822 13510648

[B5] Sinha CK Nour S Fisher R Pyloric duplication in the newborn: A rare cause of gastric outlet obstruction JIAPS 2007 12 34 35

[B6] Or MM Edwards J Neoplastic change in duplications of the alimentary tract Br J Surg 1975 62 269 273 10.1002/bjs.1800620405 1131505

